# Impact of Superior Canal Dehiscence Syndrome on Health Utility Values: A Prospective Case-Control Study

**DOI:** 10.3389/fneur.2020.552495

**Published:** 2020-10-08

**Authors:** Ibrahim Ocak, Vedat Topsakal, Paul Van de Heyning, Gilles Van Haesendonck, Cathérine Jorissen, Raymond van de Berg, Olivier M. Vanderveken, Vincent Van Rompaey

**Affiliations:** ^1^Department Otorhinolaryngology & Head and Neck Surgery, Antwerp University Hospital, Antwerp, Belgium; ^2^Translational Neurosciences, Faculty of Medicine and Health Sciences, University of Antwerp, Antwerp, Belgium; ^3^Division of Balance Disorders, Department of Otorhinolaryngology and Head and Neck Surgery, Maastricht University Medical Center, Maastricht, Netherlands; ^4^Faculty of Physics, Tomsk State University, Tomsk, Russia

**Keywords:** vestibular system, autophony, health-realeted quality of life, labyrinth diseases, vertigo

## Abstract

**Introduction:** Superior canal dehiscence syndrome (SCDS) is a condition characterized by a defect in the bone overlying the superior semicircular canal, creating a third mobile window into the inner ear. Patients can experience disabling symptoms and opt for surgical management. Limited data are available on the impact of SCDS on health-related quality of life (HRQoL) and disease-specific HRQoL more specifically.

**Objective:** To perform a prospective analysis on generic HRQoL in SCDS patients compared to healthy age-matched controls.

**Methods:** A prospective study was performed on patients diagnosed with SCDS and who did not undergo reconstructive surgery yet. Patients were recruited between November 2017 and January 2020 and asked to complete the Health Utility Index (HUI) Mark 2 (HUI2)/Mark 3 (HUI3) questionnaire. For the control group, age-matched participants without otovestibular pathology or other chronic pathology were recruited. The multi-attribute utility function (MAUF) score was calculated for the HUI2 and HUI3. Results of both groups were compared using the Mann-Whitney U test.

**Results:** A total of 20 patients completed the questionnaire. Age ranged from 37 to 79 years with a mean age of 56 years (45% males and 55% females). The control group consisted of 20 participants with a mean age of 56.4 years and ranged from 37 to 82 years (35% males and 65% females). For the case group, median HUI2 MAUF score was 0.75 and median HUI3 MAUF score was 0.65. For the control group, the median scores were 0.88 and 0.86 respectively. There was a statistically significant difference for both HUI2 (*p* = 0.024) and HUI3 (*p* = 0.011). SCDS patients had a worse generic HRQoL than age-matched healthy controls. One patient with unilateral SCDS had a negative HUI3 MAUF score (−0.07), indicating a health-state worse than death.

**Conclusion:** SCDS patients have significantly lower health utility values than an age-matched control group. This confirms the negative impact of SCDS on generic HRQoL, even when using an instrument that is not designed to be disease-specific but to assess health state in general. These data can be useful to compare impact on HRQoL among diseases.

## Introduction

First described by Lloyd Minor in 1998 (1), superior canal dehiscence syndrome (SCDS) is characterized by a defect in the bony cover of the superior semicircular canal, which creates a third mobile window into the inner ear, in addition to the round and oval window (2, 3). This third window alters the physiologic inner ear mechanics and results in a hydroacoustic shunting away from the cochlea, toward the bony defect in the labyrinth, stimulating the vestibular end organs (4). SCDS also causes enhanced bone conduction thresholds, leading to an audiometric air-bone gap, with normal stapedial reflexes (3–5). These pathophysiological features explain the symptoms patients with SCDS can experience, including autophony, aural fullness, pulsatile and non-pulsatile tinnitus, bone conduction hyperacusis, imbalance and vertigo. Gaze-evoked tinnitus, hearing distortion and oscillopsia are also possible symptoms (4, 6).

Management of SCDS depends on the severity of the symptoms. In case of mild symptoms, conservative management may include avoiding symptom triggers or placement of a tympanostomy tube for patients with primarily pressure induced symptoms (7, 8). For patients with disabling symptoms, various surgical options can be offered (9–13). Surgery has not only the potential to improve specific symptoms (14–20), but it can also improve health-related quality of life (HRQoL) (21).

Limited data are available on the impact of SCDS on generic health-related quality of life (HRQoL) and even less on disease-specific HRQoL. A distinction can be made between generic and disease-specific HRQoL instruments. Disease-specific instruments measure the HRQoL for a specific illness, allowing to detect changes after medical and/or surgical treatment or over time when treating conservatively. On the contrary, generic HRQoL instruments are designed to assess the health state in general and are not designed to detect changes in HRQoL due to a specific disease. They can be used to compare HRQoL with other chronic illnesses and a healthy population, which is not possible with a disease specific HRQoL (22). They can also be used to calculate quality adjusted life years (QALYs) and to determine cost-effectiveness of medical treatments (23, 24).

An example of generic HRQoL is the Health Utility Index (HUI). HUI consists of 2 systems, the HUI mark 2 (HUI2) and HUI mark 3 (HUI3), which are complementary to each other. HUI not only measures generic HRQoL scores, but makes it also possible to calculate single-attribute scores of morbidity for each domain of functioning (25).

The aim of this study was to perform a prospective analysis on generic HRQoL in SCDS patients compared to healthy age-matched controls.

## Materials and Methods

### Study Design

Both patients with SCDS and controls received a letter of introduction, an explanation of the purpose of the study, and the Health Utility Value Mark 2/3 questionnaire in Dutch. Informed consent was obtained from each participant as part of the survey. Study approval was obtained from the ethical committee of the University Hospital Antwerp and the University of Antwerp (B30020173349).

### Study Population

The study population comprised two groups: case and control. Cases included patients diagnosed with SCDS who had not undergone surgery for SCDS (yet). The diagnosis of SCDS was based on the combination of: (1) Symptoms related to SCDS (bone conduction hyperacusis, and/or pulsatile tinnitus, and/or sound-induced vertigo/oscillopsia, and/or pressure induced vertigo/oscillopsia); (2) Low cervical vestibular evoked myogenic potentials (cVEMPs) thresholds; (3) CT scan showing dehiscence of the superior semicircular canal (3). Surgery for SCDS in the past was an exclusion criterion. Patients younger than 18 years old were also excluded. Subjects were recruited from the tertiary neurotology clinics at the Antwerp University Hospital. The control group contained age-matched healthy controls without SCDS and without ear pathology. Controls were recruited from people accompanying patients at their visit in the department of Otorhinolaryngology and Head & Neck Surgery. Participants were questioned whether they had any hearing or balance disorders or other chronic diseases. Control participants with an otovestibular and/or a chronic disease, e.g., diabetes mellitus, pulmonary disease and cardiovascular pathology, were excluded. Hypertension was not an exclusion criterion because of its high prevalence (26). For both groups, questionnaires with incomplete data were excluded from the study. Questionnaires were sent to, and, collected from both groups between November 2017 and January 2020.

### Vestibular Testing and CT Scan

All included patients underwent a cVEMP. At the University Hospital Antwerp, air-conducted 500 Hz tone bursts were delivered monoaurally via insert phones and responses were recorded with an auditory evoked potential system equipped with electromyographic software (Neuro-Audio, Difra, Belgium), with self-adhesives electrodes (Blue sensor, Ambu, Denmark) on the sternocleidomastoid muscle. The delivered high intensity auditory stimuli resulted in a typically biphasic shape. If the wave was absent at 100 dB nHL, cVEMP response was considered to be absent. A cVEMP threshold of ≤75 dB nHL (99 dB SPL) was considered to be indicative for the presence of a third mobile window.

For the detection of dehiscence of the superior semicircular canal, a high resolution CT scan (0.625 mm slice thickness) of the temporal bone with reconstructions in the plane of the superior semicircular canal was performed. The scans were interpreted by experienced radiologists.

### Health Utility Index Mark 2 (HUI2)/Mark 3 (HUI3)

**Figure 1:**
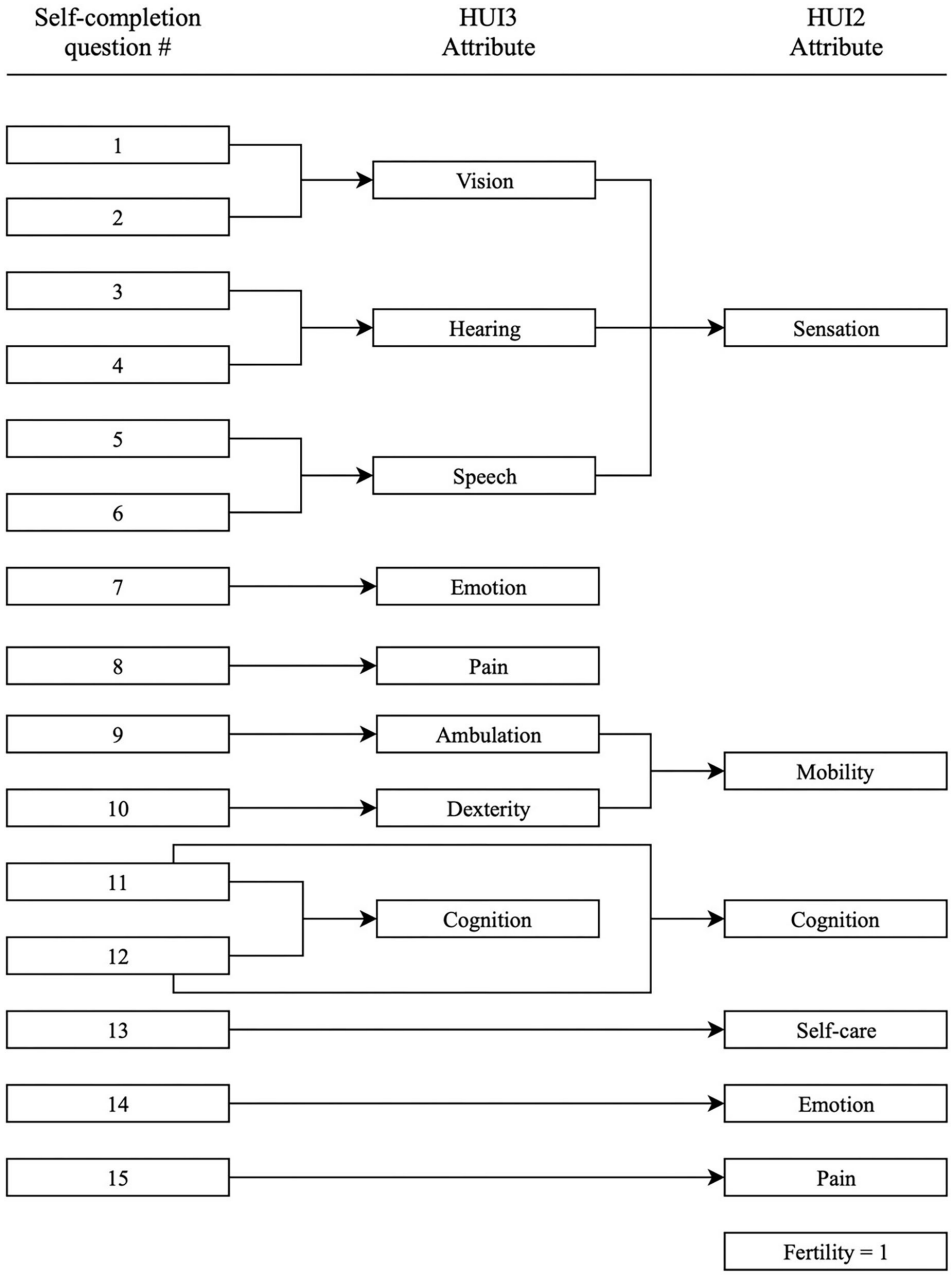
Specification of questions used to derive HUI3 and HUI2 attribute levels.

The HUI measurement system consists of a validated 15-item questionnaire for self-completion. It is designed to collect information required for classification of the participants' health status according to both the HUI2 an HUI3 classification systems. The HUI2 consists of seven domains of functioning: sensation, mobility, cognition, self-care, emotion, pain and fertility (fertility is assumed to be level 1, “able to have children with a fertile spouse,” in the HUI mark 2/mark 3 and is not asked to the subject). The HUI3 contains 8 domains of functioning, namely vision, hearing, speech, emotion, pain, ambulation, dexterity and cognition (Figure 1). Each domain has 5 or 6 levels of (dis)ability. In HUI3, sensation is divided in vision, hearing and speech, and mobility is divided in ambulation and dexterity. HUI2 contains self-care and fertility which is not implemented in HUI3. This makes HUI2 and HUI3 complementary to each. For both the HUI2 and HUI3 a multi-attribute utility function (MAUF) score can be calculated, to evaluate the general health state and HRQoL, with 1 equal to perfect health and 0 equal to death. Negative scores are possible and indicate a health state worse than death. The MAUF score can be classified in to disability categories; none, mild, moderate and severe. Different schemes are used for HUI2 and HUI3 as summed up below (25).

**Table:** 

	**HRQoL scores**	**Disability category**
HUI2	1.00	None
	0.91 through 0.99	Mild
	0.80 through 0.90	Moderate
	<0.80	Severe
HUI3	1.00	None
	0.89 through 0.99	Mild
	0.70 through 0.88	Moderate
	<0.70	Severe

### Statistical Analysis

The statistical analysis was carried out on two levels: first, a comparison of responses between the case and control groups, and secondly, analysis of responses within the case group for following variables: uni- and bilateral SCDS, and subjects opting for surgery after completing the questionnaire. All analyses were performed with IBM SPSS statistics version 25 (IBM Corp., Armonk, NY, USA).

Kolmogorov-Smirnov test was used to examine the distribution of HUI2 and HUI3 MAUF scores for both case and control group. Considering the small sample size, and normal distribution of answers (see below), the non-parametric Mann-Whitney U test was performed to compare responses between the case and control groups. The same test was also used to compare differences within the case group. A *p*-value <0.05 was used to determine statistical significance.

## Results

### Case Group

The case group consisted of 20 patients diagnosed with SCDS who had not undergone surgery for SCDS. All patients had symptoms related to SCDS, low cVEMP potentials and HRCT scan showing the dehiscence. The age ranged from 37 to 79 years with an average of 55.9 years (median 58.5 years) and standard deviation of 12.6 years. There were 11 (55%) female and 9 (45%) male patients in the case group. From the 20 patients, 17 (85.0%) had a unilateral bony defect over the superior semicircular canal, of which 7 (41.2%) were right-sided and 10 (58.8%) left-sided. Three patients (15.0%) had bilateral defects. A response rate of 100% was achieved for the case group because almost all patients completed the questionnaire directly at the clinic.

### Control Group

The control group consisted of 20 age-matched persons without otovestibular pathology or symptomatology and without any chronic disease. Age of the individuals in this group ranged from 30 to 82 years with an average of 55.9 years (median 58.5 years) and standard deviation of 12.6 years. The gender distribution was as follows: female 65.0% (*n* = 13) and male 35.0% (*n* = 7). For the control group, a high response rate of 83% was achieved. Four persons refused to participate in this study. A total of 20 healthy participants completed the questionnaire.

### HUI2 and HUI3 Multi-Attribute Utility Function (MAUF)

**Figure 2:**
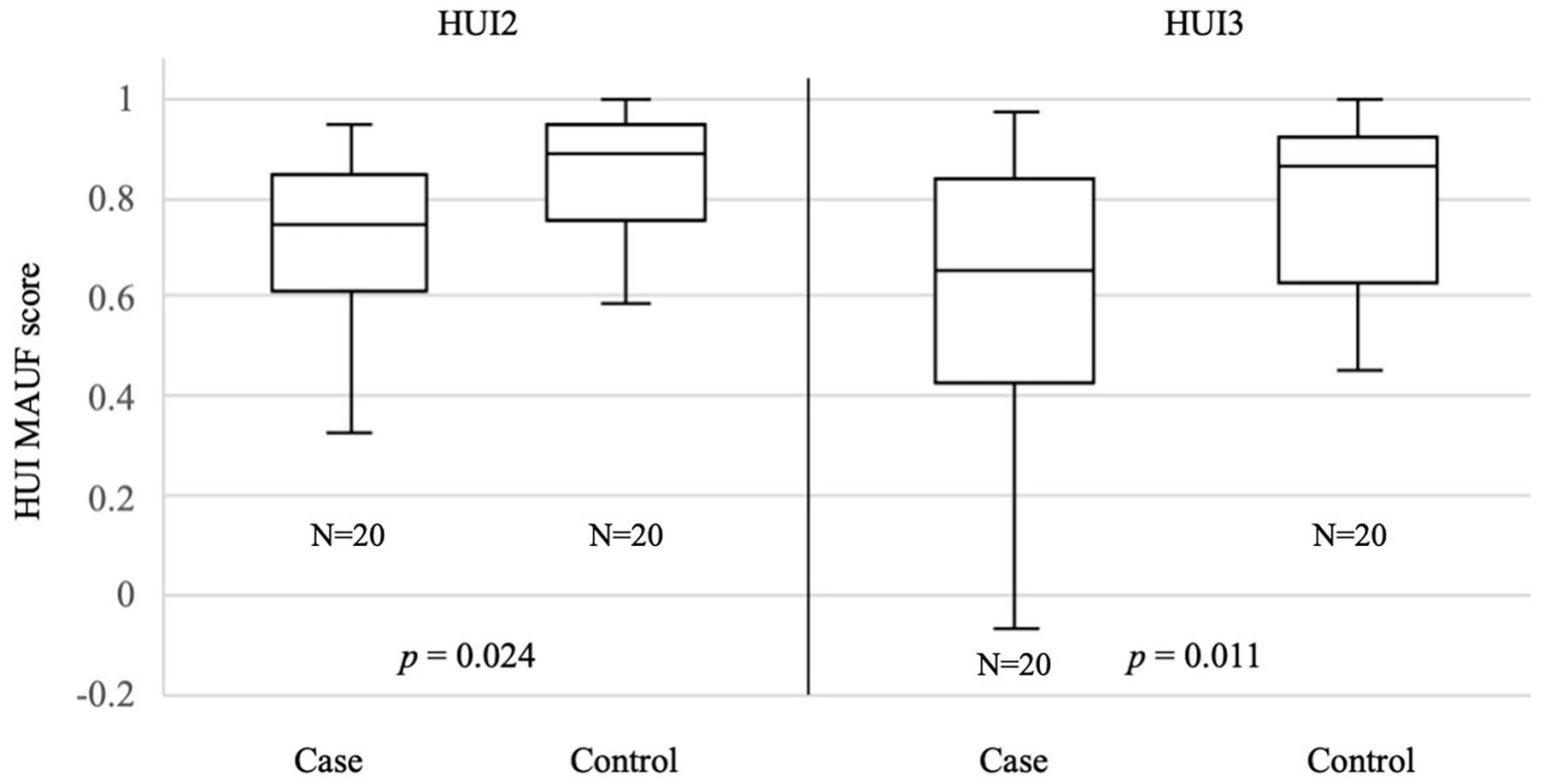
Median HUI MAUF scores for case and control groups.

Figure 2 shows the boxplot of HUI2 and HUI3 MAUF for both groups. The median HUI2 MAUF score for case group was 0.75 with a standard deviation (SD) of 0.22. For the control group, the median was 0.88 (SD= 0.14). The median HUI3 MAUF score for the case group was 0.65 (SD = 0.28). Median HUI3 MAUF score for control group was 0.86 (SD = 0.17). Comparison of case and control groups showed significantly difference for both the HUI2 MAUF (*p* = 0.024) and HUI3 MAUF (*p* = 0.011) scores. SCDS patients had a worse HRQoL than age-matched healthy controls. One patient with unilateral SCDS had a negative HUI3 MAUF score (−0.07), indicating a health-state worse than death.

The median and mean of the single attribute scores for each domain of functioning and the MAUF scores are shown in Table 1. Analysis of the single attribute scores showed significantly worse scores for HUI2 emotion (*p* = 0.023), HUI2 pain (*p* = 0.040) and HUI3 pain (*p* = 0.012) in the case group.

**Table 1:** Single and multi-attribute scores of study participants.

	**SCDS**			**Control**			
	**Mean**	**Median**	**SD**	**Mean**	**Median**	**SD**	***p*-value**
HUI2 MAUF^*^	0.70	0.75	0.22	0.84	0.88	0.14	0.024
Sensation	0.92	0.92	0.05	0.92	0.95	0.08	0.289
Mobility	1.00	1.00	0.01	0.99	1.00	0.04	0.336
Cognition	0.97	1.00	0.04	0.99	1.00	0.02	0.218
Self-care	1.00	1.00	0.01	1.00	1.00	0	0.317
Emotion	0.93	0.93	0.07	0.97	1.00	0.05	0.023
Pain	0.86	0.97	0.20	0.96	1.00	0.09	0.040
HUI3 MAUF	0.59	0.65	0.28	0.80	0.86	0.17	0.011
Vision	0.97	0.98	0.04	0.98	0.98	0.02	0.989
Hearing	0.97	1.00	0.07	0.97	1.00	0.10	0.678
Speech	0.98	1.00	0.04	0.99	1.00	0.02	0.301
Emotion	0.93	0.95	0.09	0.97	1.00	0.05	0.096
Pain	0.89	0.90	0.09	0.96	0.96	0.04	0.012
Ambulation	0.99	1.00	0.03	0.99	1.00	0.03	0.620
Dexterity	1.00	1.00	0.01	1.00	1.00	0	0.799
Cognition	0.91	1.00	0.15	0.98	1.00	0.03	0.355

* *Fertility is considered to be 1*.

*HUI, Health Utility Index; MAUF, multi-attribute utility function; SCDS, superior canal dehiscence syndrome*.

*Underlined values are p < 0.05 indicating statistical significance*.

**Figure 3:**
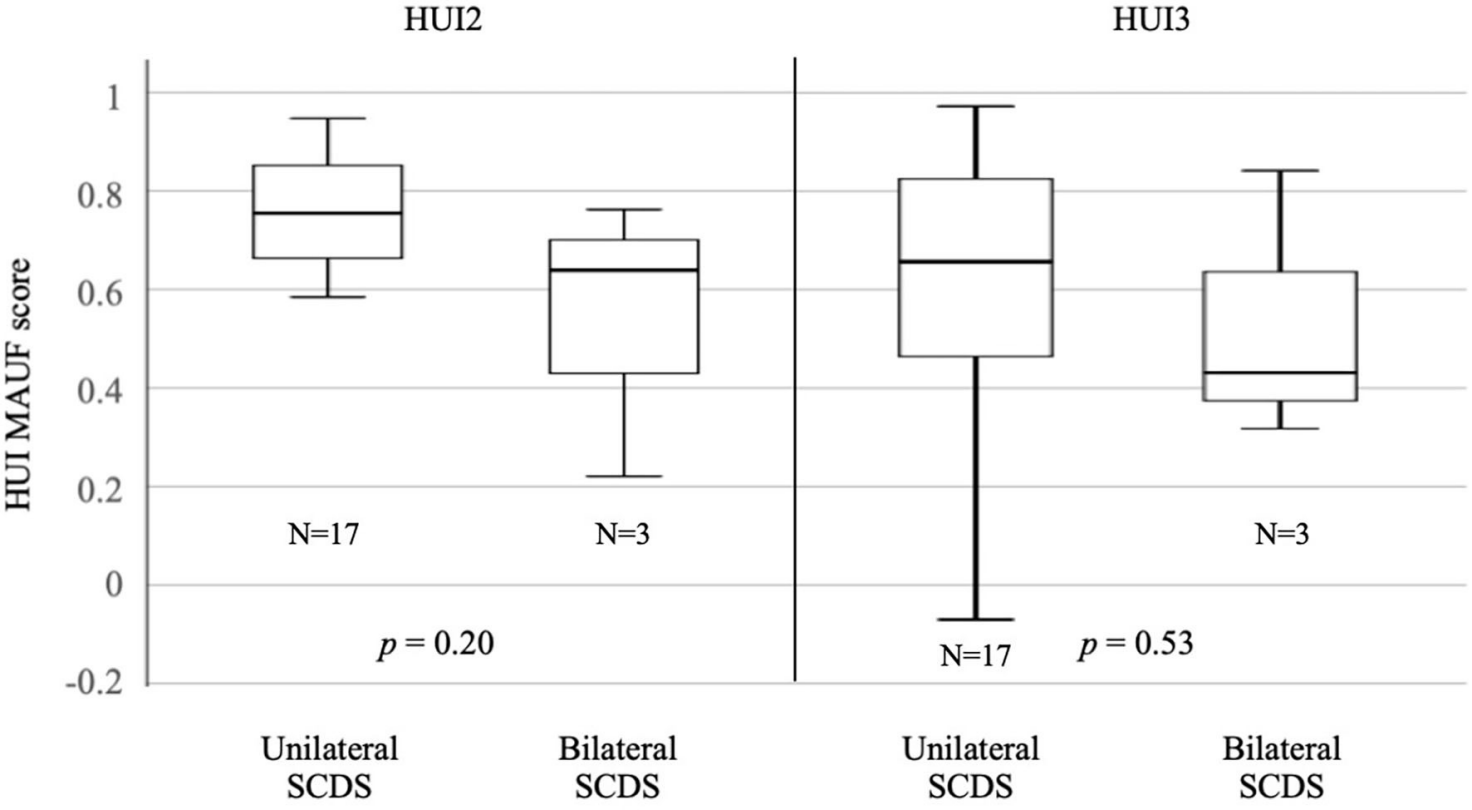
Median HUI MAUF scores for uni- vs. bilateral SCDS.

Comparison of uni- vs. bilateral SCDS and HUI2 showed median MAUF score of 0.75 (SD = 0.21) for the unilateral SCDS group, and 0.64 (SD = 0.28) for the bilateral SCDS group. The median HUI3 MAUF score for the unilateral group was 0.66 (SD = 0.29) and 0.43 (SD = 0.28) for the bilateral group. There was no statistically significant difference for HUI2 (*p* = 0.20) and HUI3 (*p* = 0.53) scores for uni- vs. bilateral SCDS (Figure 3).

**Figure 4:**
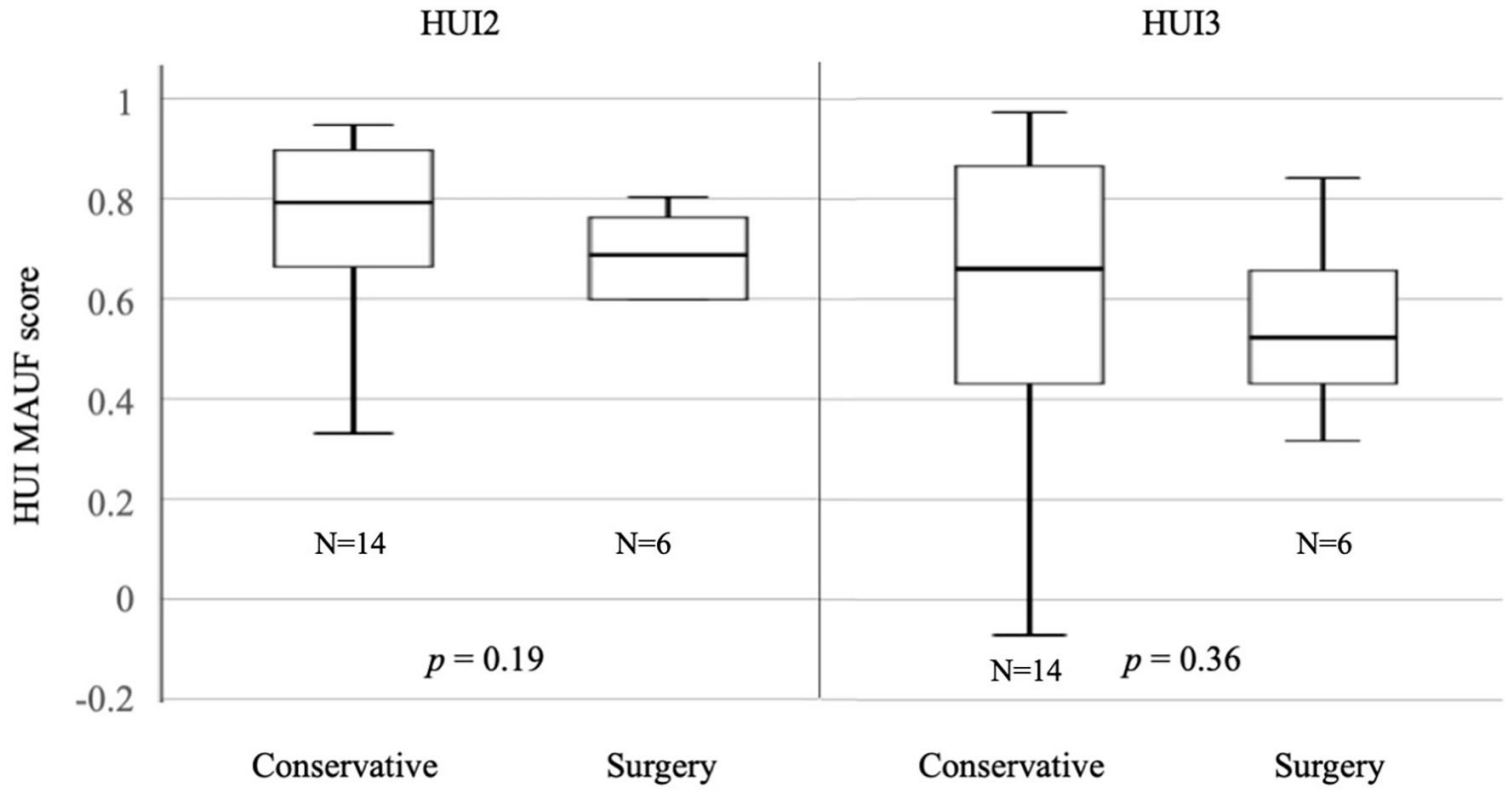
Median HUI2 MAUF scores for conservative vs. surgical approach.

After completing the questionnaire, 6 patients opted for surgery. Comparison of surgery vs. conservative approach within the case group showed no statistically significant differences for HUI2 (*p* = 0.19) and HUI3 (*p* = 0.36). The median HUI2 MAUF score was 0.79 (SD = 0.22) and median HUI3 MAUF score was 0.66 (SD = 0.32) for the conservative group. The surgery group had a median HUI2 MAUF score 0.69 (SD = 0.21) and a median HUI3 MAUF score of 0.53, with a SD of 0.19 (Figure 4). It is important to mention that all the patients completed the questionnaire prior to any treatment. A comparison of pre- and postoperative was not performed.

## Discussion

The aim of this study was to perform a prospective analysis on generic HRQoL in SCDS patients compared to healthy age-matched controls. Patients with SCDS can experience a wide variety of symptoms. If the symptoms are disabling and have a negative impact on the HRQoL, surgery can be offered to patients. However, limited data are available on the impact of SCDS on generic HRQoL. Generic HRQoL can, for example, be used to calculate quality adjusted life years (QALYs) and to determine cost-effectiveness of medical treatments, like transmastoid vs. middle cranial fossa approach or plugging vs. resurfacing for SCDS repair (27). Our data set can also be used to calculate QALYs and to compare with other studies. However, a more reliable comparison can be made with a larger data set (multicentric for example).

Comparison of case and control group revealed significant difference in HUI2 and HUI3 MAUF scores, with lower scores for the case group. Analysis of the single attribute levels showed worse scores for HUI2 emotion (*p* = 0.023), HUI2 pain (*p* = 0.040) and HUI3 pain (*p* = 0.012) in the case group. Lower scores for pain may be explained by hyperacusis but further research is needed. Patients with SCDS can also experience depression, as shown in the study of Wackym et al. They investigated the cognitive and neurobehavioral outcome before and after surgical repair of otic capsule dehiscence. Preoperative completion of the Beck Depression Inventory-II showed mild depression, which improved after surgery (28). This can explain the negative impact on the attribute “emotion” in our study population.

The subgroup analysis of the case group did not reveal any statistically significant differences comparing bilateral to unilateral SCDS patients and patients who opted for surgery compared to patients who chose a conservative approach. Surgery has the potential to improve symptoms such as autophony, and pulsatile tinnitus (14, 15, 29). However, these symptoms are not measured by HUI, because it is a generic HRQoL instrument. This could (partially) explain why there was no significant difference in HUI scores between the patients who opted for surgery and the patients who chose a conservative approach. It is important to mention that all the patients completed the questionnaire prior to any treatment. A comparison of pre- and postoperative was not performed.

Analysis of health utility values (HUV) after surgery for SCDS was performed by Remenschneider et al. They investigated the HUV in 51 patients with SCDS. The HUV was measured by Short-Form 6 Dimension Questionnaire. Twenty-three of 51 patients opted for surgery. There was no significant difference between the operated and non-operated group preoperatively. We had a similar finding for the preoperative comparison of the HUI values between the conservative and surgery group, however the sample size in our study was lower. Analysis of HUV after surgery showed a significant improvement of the HUV (21). Allsopp et al. investigated QoL outcomes after transmastoid plugging of SCDS retrospectively. Generic HRQoL was calculated by the Glasgow Benefit Inventory (GBI). Ten patients were enrolled in the study. Postoperative GBI values were significantly better (30). These results indicate that surgery is a good option which can increase the HRQoL in patients with SCDS. In this study, postoperative HUI values were not compared with preoperative results, because postoperative questionnaires were not (yet) filled by the patients who opted for surgery.

Generic HRQoL can also be used to compare HRQoL among different pathologies. Sun et al. compared HRQoL, measured with the dizziness handicap index and HUI3, in 15 patients with bilateral vestibular deficiency (BVD), 22 patients with unilateral vestibular deficiency (UVD) and 23 healthy controls. BVD patients had a significantly decreased HRQoL compared to UVD and healthy controls. The mean HUI3 MAUF score was 0.39 (SD = 0.34) for the BVD, 0.63 (SD = 0.26) for the UVD and 0.94 (SD = 0.09) for the control group (31). Our data demonstrated a median HUI3 MAUF score of 0.65 (SD = 0.28) for the SCDS patients and 0.86 (SD = 0.17) for the control group as shown in Figure 2. Patients with SCDS had a worse HUI3 score with statistically significant difference compared to healthy controls. Both BVD and SCDS can have a negative impact on HRQoL and therefore surgical treatments might be considered or developed for SCDS and BVD respectively, in case of disabling symptoms (32, 33).

Carlsson et al. investigated the QoL in 369 patients with sudden sensorineural hearing loss (SSHL). QoL was measured by the EuroQoL 5D, problems impact rating scale and hospital anxiety and depression scale. In patients with tinnitus and remaining vertigo after SSHL, a significant negative impact on all three QoL measurements was found (34).

The major limitation of this study is the rather low sample size. Sample size was even lower when performing subgroup analysis. There were only three patients with bilateral SCDS and six patients opting for surgery after completing the questionnaire. This makes statistical analysis difficult and no statistically significant differences were calculated in the subgroup analysis.

Even though our data highlights that SCDS can have an impact on the generic HRQoL, the syndrome can cause a wide range of symptoms and clinical presentation can be different for each case. It can also be difficult for patients to spontaneously mention some of the “odd” symptoms, like “hearing the eyeballs move.” This points to the need of an evidence-based disease-specific patient-reported outcome measure (PROM) (6). With such a measurement, the prevalence and severity of the symptoms can be evaluated, and the impact on HRQoL might be estimated (35).

## Conclusion

SCDS patients have significantly lower generic HRQoL scores, measured with HUI2 and HUI3, than an age-matched control group. This confirms the negative impact of SCDS on generic HRQoL, even when using an instrument that is not designed to assess disease-specific HRQoL but to assess health state in general. These data can be useful to compare impact on HRQoL among diseases. In addition, there is a need for a disease-specific PROM for SCDS in order to properly investigate the prevalence and severity of symptoms SCDS patients are experiencing. Such a measurement can also be useful to evaluate treatment more objectively over time, than only history taking.

## Data Availability Statement

The raw data supporting the conclusions of this article will be made available by the authors, without undue reservation.

## Ethics Statement

The studies involving human participants were reviewed and approved by Antwerp University Hospital/University of Antwerp Ethics Committee. The patients/participants provided their written informed consent to participate in this study.

## Author Contributions

VVR conceived the idea and developed the theory of the research. IO carried out the study and performed the statistical analysis. Database of patients was started by GVH and updated by VVR and IO. Controls were recruited by IO. Questionnaires were processed and analyzed by IO. CJ helped with the writing of the cVEMP and HUI methodology. The manuscript was reviewed by VT, PVDH, GVH, CJ, OV, RVDB, VVR and IO. All authors contributed to the article and approved the submitted version.

## Conflict of Interest

The authors declare that the research was conducted in the absence of any commercial or financial relationships that could be construed as a potential conflict of interest.
